# EXERCISE THERAPY: AN ALTERNATIVE APPROACH TO MANAGING PAIN IN GERIATRIC PATIENTS

**DOI:** 10.1093/geroni/igz038.1494

**Published:** 2019-11-08

**Authors:** Lydia In

**Affiliations:** University of Southern California, Los Angeles, California, United States

## Abstract

Physical therapy improves the physical function and strength of older adults. Exercise decreases pain associated with osteoarthritis of the knee and hip, while improving well-being and physical function in patients with fibromyalgia. Current CDC guidelines for chronic pain management recommend exercise therapy as one of the non-pharmacologic strategies to employ. Based on a patient’s personal goals, physical therapists, experts in human movement, design individualized care plans, provide appropriate interventions, and modify treatment as necessary to optimize or maintain function, improve quality of life, and delay dependency and need of care. When PTs work with patients in pain, they focus on the movement patterns that may contribute to the pain. PTs also evaluate risk factors for pain to help prevent future pain issues. Strategies employed by physical therapists will be discussed, and well as ways to integrate exercise into a holistic and multi-disciplinary pain management approach.

